# Recurrent T-lymphoblastic lymphoma in a pediatric patient with bilateral breast and skin involvement: A rare clinical presentation

**DOI:** 10.1097/MS9.0000000000003506

**Published:** 2025-06-25

**Authors:** Şule Çalışkan Kamış, Begül Yağcı

**Affiliations:** Department of Pediatric Hematology and Oncology, University of Health Sciences, Adana Faculty of Medicine, Adana City Education and Research Hospital, Adana, Turkey

**Keywords:** case report, skin manifestations, T-lymphoblastic lymphoma

## Abstract

**Introduction and importance:**

T-lymphoblastic lymphoma (T-LBL) is a rare and aggressive subtype of precursor T-cell lymphomas, often presenting with lymph node, bone marrow, and mediastinal involvement. Cutaneous involvement is uncommon but associated with a poor prognosis.

**Case presentation:**

We report the case of a 17-year-old female with relapsed T-LBL presenting with bilateral breast masses, confirmed by biopsy and immunohistochemistry. Initial treatment with a BFM ALL chemotherapy protocol resulted in partial response, but disease recurrence with cutaneous and cervical lymph node involvement necessitated further treatment with the ALLIC BFM 2016 Relapse protocol. The disease course was complicated by avascular necrosis requiring hip arthroplasty, and follow-up PET/CT scans revealed further extranodal involvement, including the skin.

**Discussion:**

Extranodal involvement in peripheral T-cell lymphomas (PTCL), such as T-LBL, poses significant challenges to clinical management due to its association with aggressive disease and poor prognosis. Standard CHOP regimens often result in short-term remission with high relapse rates. Emerging therapies, including HDAC inhibitors, proteasome inhibitors, and immunotherapies, show promise but require further study to address tumor heterogeneity and optimize efficacy.

**Conclusion:**

This case underscores the critical role of early diagnosis and effective multidisciplinary management in relapsed T-LBL with skin involvement. Further research is needed to develop personalized therapeutic strategies to improve outcomes in this rare and aggressive lymphoma subtype.

## Introduction

Primary cutaneous lymphomas (PCLs) represent a heterogeneous group of extranodal non-Hodgkin lymphomas primarily involving the skin without initial extracutaneous disease. Although PCLs predominantly affect adults, recent studies have highlighted their occurrence in the pediatric population as well^[^1^]^. However, data regarding pediatric PCLs remain limited compared to adult cases, and the clinical behavior may differ from adult presentations^[^1^]^.

T-lymphoblastic lymphoma (T-LBL) is a rare and aggressive lymphoma that arises from precursor T lymphocytes, accounting for about 2% of lymphoid leukemias^[^2^]^. Skin involvement can be an initial sign of the disease and aids in accurate diagnosis. However, it is associated with a poor prognosis. LBL is more commonly observed in late childhood and young adult males, with a second incidence peak in individuals aged 60–70 years^[^3^]^. Acute T-lymphoblastic leukemia/lymphoma and peripheral T-cell lymphoma (PTCL) are rarely seen together^[^4^]^. T-LBL typically presents with signs of lymph node, bone marrow, and mediastinal mass invasion, but in rare cases, it can also manifest with skin involvement. A review of cutaneous T-LBL cases showed frequent expression of tumor markers such as TdT (100%), CD3 (100%), CD4 (59.1%), and CD99 (40.9%)^[^5^]^. In the treatment of T-LBL, chemotherapy regimens similar to those used for ALL protocols are typically applied. However, in cases with skin involvement, the response to treatment is generally lower, and the prognosis is poor. This underscores the critical importance of early diagnosis^[^6^]^. In the literature, data on the efficacy of immunotherapies in T-LBL cases with skin involvement are limited. Emerging targeted therapies and immune checkpoint inhibitors are being investigated as promising treatment options in this rare disease group.

Immunotherapies such as alemtuzumab and mogamulizumab have shown promising results in PTCL treatment^[^7^]^. Alemtuzumab has been effective when combined with CHOP (cyclophosphamide, doxorubicin, vincristine, prednisone) chemotherapy, although its use has been limited due to the risk of infections. Mogamulizumab, on the other hand, has been effective in relapsed/refractory PTCL and cutaneous T-cell lymphoma (CTCL) patients expressing CCR4, with manageable side effects. These therapies hold potential when combined with chemotherapy^[^8^]^.

The aim of this case report is to highlight the rare clinical presentation of relapsed T-LBL with bilateral breast and skin involvement in a pediatric patient, and to emphasize the importance of early diagnosis and multidisciplinary management in such unusual manifestations. Timely diagnosis can significantly improve the chances of effective treatment and better prognosis.

## Case presentation

A 17-year-old female patient presented palpable masses in both breasts and no systemic symptoms such as fever, weight loss, or night sweats. She had a significant medical history of stage 3 lymphoblastic lymphoma (LBL), diagnosed 10 years prior, for which she had been treated at another center with the Berlin-Frankfurt-Münster Non-Hodgkin Lymphoma (BFM-NHL) protocol. She had completed four cycles of the AA-BB protocol, achieving the cumulative doses for etoposide (VP-16) and adriamycin (doxorubicin).

On physical examination, firm, mobile masses were palpated bilaterally in the breasts without associated axillary lymphadenopathy.

Breast ultrasonography revealed a 2.5 mm cyst with anechoic content in the right breast. Additionally, a 9 × 16 mm lobulated hypoechoic lesion was noted in the right breast, approximately 1 cm from the nipple. In the left breast, a 5 × 9 mm lobulated hypoechoic lesion was observed, 3 cm from the nipple, along with various other hypoechoic solid lesions in different areas.

A tru-cut biopsy of the 9 × 16 mm hypoechoic lesion in the right breast revealed pathology consistent with lymphoblastic lymphoma. Immunohistochemical staining demonstrated that the neoplastic cells were positive for CD34, terminal deoxynucleotidyl transferase (TdT), CD3, CD5, CD7, and B-cell lymphoma 2 (bcl2), with focal positivity for CD4 and CD8, while being negative for CD20, CD79a, bcl1 (B-cell lymphoma 1), B-cell lymphoma 6 (bcl6), CD30, and CD56. The Ki67 proliferation index was 85%.

Positron emission tomography/computed tomography (PET/CT) imaging demonstrated multiple hypermetabolic nodular lesions in the bilateral breast tissues, with more pronounced activity on the left side. Mild-to-moderate metabolic activity was observed in the bilateral cervical lymph nodes, and a hypermetabolic cystic lesion was identified in the left adnexal area. Other findings included focal hypermetabolic lesions in the colon and other soft tissue areas.

Bone marrow aspiration, flow cytometry, and biopsy results were normal, and cerebrospinal fluid (CSF) examination revealed no cells suggestive of central nervous system (CNS) involvement.

Additionally, conventional cytogenetic analysis and flow cytometry studies were performed on the bone marrow samples, revealing no cytogenetic abnormalities or evidence of clonal proliferation.

Based on these findings, the patient was diagnosed with relapsed T-LBL localized to the breasts and cervical lymph nodes. Chemotherapy following the BFM ALL protocol was initiated.

During treatment, the patient was evaluated before transitioning to oral maintenance chemotherapy, with no indication for radiotherapy. Follow-up PET/CT scans showed partial regression in the size and metabolic activity of the breast lesions and cervical lymph nodes. Diffuse hypermetabolism observed in the right palatine tonsil and bilateral adnexal cystic lesions were interpreted as inflammatory or reactive changes, considered false-positive findings.

**HIGHLIGHTS**

- To our knowledge, first pediatric t-lbl case with bilateral breast and skin involvement.
- Extranodal skin and breast involvement posed significant diagnostic challenges.
- Immunohistochemistry confirmed aggressive relapse requiring reinduction therapy.
- Multidisciplinary management improved care of rare extranodal lymphoma sites.
- Early biopsy is essential in atypical pediatric lymphoma relapse presentations.

While on maintenance therapy, the patient developed right thigh pain. Magnetic resonance imaging (MRI) of the hip revealed a hypointense area on T1-weighted images and hyperintense signal on T2-weighted images at the right femoral head and neck, compatible with avascular necrosis. Bone scintigraphy confirmed the diagnosis, and the patient subsequently underwent a total right hip arthroplasty.

**Figure 1.**
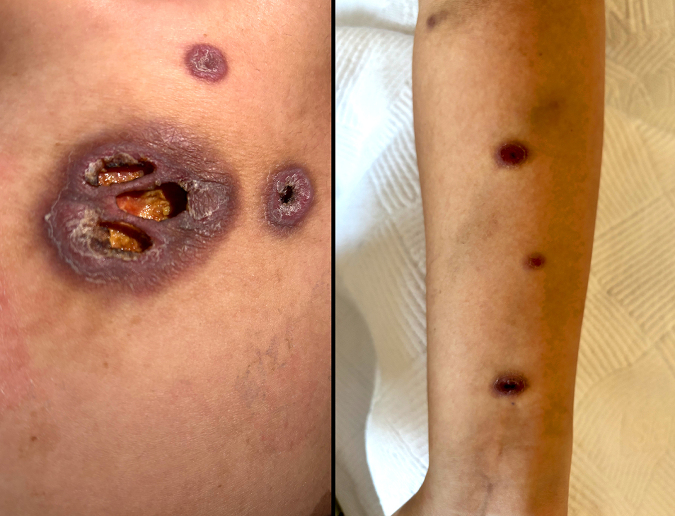
Widespread furuncular skin lesions observed during relapse of T-lymphoblastic lymphoma (T-LBL). The lesions were distributed over the trunk and extremities and initially mimicked benign infectious or inflammatory conditions. Histopathological examination of skin biopsies confirmed infiltration by T-LBL cells, highlighting the importance of considering lymphoma relapse in the differential diagnosis of atypical skin eruptions in patients with a prior history of T-LBL.

After completing and discontinuing oral maintenance therapy, the patient remained under observation for 2 years without treatment. She subsequently presented with widespread furuncular lesions and back pain (Fig. 1).

Right shoulder MRI revealed minimal effusion in the superior subscapular recess, while abdominal and breast ultrasound findings were within normal limits. However, repeat PET/CT imaging demonstrated conglomerated lymph nodes with intense metabolic activity in the left cervical chain and metabolically active soft tissue lesions in the neck and pelvic regions.

A cervical lymph node biopsy from the left cervical region confirmed T-LBL, with immunohistochemistry showing positive staining for CD7, CD3, and CD34, and negative staining for CD20 and CD5, with a Ki67 proliferation index of 70%. A skin biopsy also reported infiltration of T-LBL in the skin and subcutaneous tissue, with positivity for CD7, CD3, and CD34, and negativity for CD20, CD5, and CD30.

Following these findings, reinduction chemotherapy was initiated according to the ALLIC BFM 2016 Relapse protocol.

## Discussion

Peripheral T-cell lymphoma (PTCL) is typically a nodal lymphoma but can frequently exhibit extranodal involvement, which significantly impacts the disease course and clinical management. Extranodal involvement is often associated with a more aggressive disease course and poorer prognosis. The most commonly affected extranodal sites include the skin and gastrointestinal tract, further complicating the management of PTCL and highlighting the need for a comprehensive and individualized treatment approach^[^9^]^.

The standard frontline treatment for PTCL is the CHOP regimen (cyclophosphamide, doxorubicin, vincristine, prednisone), which achieves remission in approximately 50–65% of cases. However, these responses are often short-lived, and relapse rates are high, underscoring the necessity for alternative therapeutic strategies. High-dose chemotherapy followed by hematopoietic stem cell transplantation has been suggested to improve prognosis, although these approaches are limited to patients who can tolerate intensive therapy^[^10,11^]^.

In recent years, the importance of developing novel treatment strategies for PTCL has become increasingly evident. Preclinical and clinical studies have demonstrated that combinations of histone deacetylase (HDAC) inhibitors, proteasome inhibitors, pralatrexate, and bortezomib show synergistic effects in the treatment of PTCL. Nevertheless, treatment responses may vary depending on the genetic and biological characteristics of individual patients, emphasizing the need for more targeted therapeutic approaches^[^12,13^]^.

Immunotherapies, particularly immune checkpoint inhibitors and chimeric antigen receptor (CAR) T-cell therapies, represent promising advancements in PTCL management^[^14,15^]^. However, several challenges persist, including tumor heterogeneity, immune evasion mechanisms, and difficulties in identifying suitable therapeutic targets^[^16,17^]^. Advanced molecular analyses and a deeper understanding of intracellular signaling pathways play a crucial role in the development of more effective and personalized treatment strategies^[^18,19^]^.

Strategies targeting the tumor microenvironment (TME) also hold significant promise by enhancing immune cell infiltration, disrupting stromal cell interactions, or reprogramming tumor-associated macrophages to improve PTCL treatment outcomes^[^20,21^]^.

Relapsed T-lymphoblastic lymphoma (T-LBL) with extranodal involvement, particularly involving the skin and breast, poses notable diagnostic challenges. Such atypical presentations may initially mimic benign conditions such as infections, inflammatory breast disease, or primary cutaneous neoplasms, potentially leading to diagnostic delays. In our case, bilateral breast masses and furuncular skin lesions initially raised suspicion for benign processes, delaying consideration of lymphoma relapse. Accurate diagnosis required high clinical suspicion, comprehensive imaging, histopathological evaluation, and immunohistochemical studies. These findings highlight the importance of early biopsy and multidisciplinary evaluation in patients with unusual presentations and a prior history of T-LBL.

Future research is expected to focus on gaining a deeper understanding of the molecular and genetic underpinnings of PTCL, which may pave the way for more targeted and effective therapeutic protocols. These approaches have the potential to enhance patient responses, prolong remission periods, and ultimately improve survival outcomes.

This case report has been written in accordance with the scare 2023 guidelines^[^22^]^.

## Conclusion

In conclusion, this case highlights the critical importance of early diagnosis and multidisciplinary management in relapsed T-lymphoblastic lymphoma with skin involvement. Although the treatment of peripheral T-cell lymphoma (PTCL) remains challenging, recent advances in molecular diagnostics, targeted therapies, and immunotherapy offer promising strategies for improving clinical outcomes. Future research should focus on personalized therapeutic approaches and targeting the tumor microenvironment to enhance survival rates and quality of life in patients with this rare and aggressive lymphoma subtype.

## Data Availability

Data and materials will be made available upon reasonable request and with the author’s approval.

## Research registration unique identifying number (UIN)

This study is a case report, and according to the World Medical Association’s Declaration of Helsinki (2013), registration in a publicly accessible database is not required for single case studies. Ethical approval was not obtained, as this is a retrospective case report utilizing existing clinical data. The study was conducted in accordance with the ethical principles outlined in the Declaration of Helsinki.
